# Understanding attitudes and obstacles to vaccination against COVID-19 in patients with primary immunodeficiency

**DOI:** 10.1186/s13223-022-00679-x

**Published:** 2022-05-09

**Authors:** Babak Aberumand, Whitney Ayoub Goulstone, Stephen Betschel

**Affiliations:** 1 Division of Allergy & Immunology, Department of Medicine, University of Toronto, 30 Bond St., Toronto, ON M5B 1W8 Canada; 2 Canadian Immunodeficiencies Patient Organization, Victoria, BC Canada; 3 Division of Allergy & Immunology, Department of Medicine, St. Michael’s Hospital, Toronto, ON Canada

**Keywords:** COVID-19, SARS-CoV-2, Immune response, Immunocompromised, Immunosuppressed, Primary immunodeficiency, Vaccination, Vaccine hesitancy

## Abstract

**Background:**

Patients with primary immunodeficiency (PID) are at increased risk for infections such as SARS-CoV-2 (COVID-19), due to the nature of their diseases and being immunocompromised. At this time, four vaccines against COVID-19 (Pfizer-BioNtech’s Comirnaty^®^, Moderna’s Spikevax^®^, AstraZeneca’s Vaxzevria^®^, Johnson & Johnson’s Janssen^®^) have been approved for use by Health Canada. Due to the novelty of these vaccines, clinical studies in patients with PID are ongoing. Despite limited evidence, Canada’s National Advisory Committee on Immunization (NACI) recommend that patients with PID without any contraindications should be vaccinated with any of the approved vaccines as the potential benefits of being immunized against the virus likely outweigh the risks of contracting a severe infection. The aim of this study was to understand the perceptions regarding COVID-19 vaccination among patients with PID and to identify specific factors related to vaccine hesitancy.

**Methods:**

The Canadian Immunodeficiencies Patient Organization (CIPO) conducted an online survey of its members to evaluate uptake of the COVID-19 vaccines by patients with PID. Data was collected using a self-administered online questionnaire. The survey was conducted between March and April 2021.

**Results:**

At the time of survey, among 370 respondents who had not received the COVID-19 vaccine, 302 respondents (81.6%) indicated they were very or somewhat likely to get vaccinated against COVID-19; and 68 respondents (18.4%) indicated they were somewhat or very unlikely, undecided, or not planning to get vaccinated. A large majority of respondents indicated they had a diagnosis of PID (67.8%) and/or specified their type of PID (27.7%). The most common reason for vaccine hesitancy was primarily due to uncertainty about immune response given an underlying immunodeficiency. Other concerns included unknown long-term side effects of COVID-19 vaccination, pre-existing history of allergic reactions, limited amount of data, lack of investigation of safety and effectiveness of COVID-19 vaccines in those with medical conditions, and skepticism of the underlying science and/or the medical system.

**Conclusions:**

The results point to the importance of ongoing patient outreach, education, and up-to-date information on the rapidly evolving scientific knowledge and evidence on COVID-19 relevant to the PID community, from clinical trials to real-world evidence and observational studies.

## Background

Primary immunodeficiency disorder refers to a group of over 400 genetic disorders due to gene defects in specific cells and proteins of the immune system. This is characterized by poor or dysregulated function in one or more components of the immune system [1, 2]. The disorders have different and diverse underlying phenotypes leading to infection, malignancy, allergy, auto-immunity, and auto-inflammation. Patients with PID are prone to viral, bacterial, and fungal infections, especially those involving the sinopulmonary tract. These are often recurrent, persistent, or severe infections. Due to the nature of their disorder, patients with PID are thought to have a poor outcome with respect to morbidity and mortality should they contract COVID-19 [1, 3]. However, this may not be characteristic of all patients with PID, but rather specific to a certain subset, especially those that are younger and those with reduced type I interferon signaling [4–6].

At this time, four vaccines against COVID-19 (Pfizer-BioNtech’s Comirnaty^®^, Moderna’s Spikevax^®^, AstraZeneca’s Vaxzevria^®^, Johnson & Johnson’s Janssen^®^) have been approved for use by Health Canada. Due to the novelty of these messenger ribonucleic acid (mRNA—Pfizer-BioNtech’s Comirnaty^®^, Moderna’s Spikevax^®^) and viral vector-based (AstraZeneca’s Vaxzevria^®^, Johnson & Johnson’s Janssen^®^) vaccines against COVID-19, they have not been extensively studied in patients with PID since the vaccination campaign commenced. Although studies are currently limited, there does appear some evidence to suggest that patients with PID are able to mount at least a partial if not full response to the mRNA COVID-19 vaccine [7–9]. At the time of this study, the recommendations of Canada’s National Advisory Committee on Immunization (NACI) were that patients with PID who do not have any contradictions to the vaccine should be offered any of the four COVID-19 vaccines, as the potential benefits of being immunized against SARS-CoV-2 likely outweigh the risk of severe COVID-19 infection [10].

Global rapid review studies in Organisation for Economic Co-operation and Development (OECD) countries, including Canada, show that patients with severe immunodeficiency, whether primary or secondary, who have contracted COVID-19 tend to suffer far worse outcomes than the general population [10]. Recent Canadian surveillance data contributed by the Alberta Research Centre for Health Evidence (ARCHE) indicate that compared to the general population, a higher proportion of the immunocompromised population are hospitalized and admitted to the intensive care unit due to COVID-19. The review found limited (low/moderate certainty) evidence that patients with severe primary or secondary immunodeficiency have at least twofold increased risk of hospitalization; and strong (moderate/high certainty) evidence of at least twofold increased risk of mortality for some specific medical conditions such as myasthenia gravis, which is often associated with selective IgM deficiency. The degree of immunodeficiency in immunocompromised patients is variable depending on the underlying condition, the progression of disease, and use of medications that suppress immune function [10, 11]. These factors, together with the rapidly evolving scientific knowledge of COVID-19, augment the complexities of COVID-19 vaccination and management of care for patients with PID.

Throughout the course of the pandemic, CIPO has provided the PID community in Canada with COVID-19 information, updates, and guidance in accordance with recommendations issued by NACI and the Canadian Society of Allergy and Clinical Immunology (CSACI) [11, 12]. In March 2021, CSACI published guidance advising that COVID-19 vaccines should be offered to immunocompromised patients if the benefit is deemed to outweigh any potential risks of vaccination; and that assessment by a clinical immunologist and allergist is warranted in any individual with a suspected allergy to a COVID-19 vaccine or any of its components. Assessment by a clinical immunologist and allergist is not required for individuals with a history of unrelated allergies [12]. In June 2021, NACI published updated recommendations for immunocompromised populations, stating that emerging data suggest that all authorized vaccines offer protection against hospitalization and death from COVID-19. It made a strong recommendation for the preferential use of mRNA COVID-19 vaccines in all authorized age groups, and a discretionary recommendation for the use of viral-vector COVID-19 vaccines for individuals when other authorized COVID-19 vaccines are contraindicated or inaccessible. NACI cautioned that there is uncertainty in the evidence of advantages and disadvantages of the use of viral-vector COVID-19 vaccines for eligible populations in Canada due to the risk of a rare but serious adverse event known as vaccine-induced thrombotic thrombocytopenia (VITT) [10].

The aim of this study was to understand the perceptions regarding uptake in the COVID-19 vaccines by patients with PID in Canada and to identify the factors related to vaccine hesitancy. As defined by the World Health Organization’s Strategic Advisory Group of Experts (SAGE) on Immunization as “delay in acceptance or refusal of vaccination despite availability of vaccination services” [13]. The purpose of characterizing the nature and scale of vaccine hesitancy issues among patients with PID was to better inform the development of appropriate strategies to address the concerns expressed, minimize vaccine hesitancy, and sustain confidence in vaccination.

## Methods

CIPO conducted a national survey to evaluate uptake of the COVID-19 vaccines in patients with PID in Canada. Data was collected using the SurveyMonkey Internet-based platform. The self-administered online questionnaire included questions on whether or not respondents had received a COVID-19 vaccine, whether or not they plan on being vaccinated, and reasons for being hesitant or not intending to get vaccinated. The survey also included questions related to perspectives and behaviours regarding vaccination against influenza, for comparison with the COVID-19 data. It is important to note that respondents were permitted to skip any questions that were not applicable to them, or that they did not wish to answer. For open-ended questions with multiple answer choices, respondents were instructed to select all options that applied.

The survey was publicized and disseminated primarily through an email campaign directed at CIPO’s membership, which includes patients with PID, caregivers, patient organizations, and healthcare providers. A total of 1131 members were contacted via email. It was also promoted through CIPO’s social media networks including its website and through its medical network. The survey took place from March 24 to April 7, 2021.

## Results

**Fig. 1:**
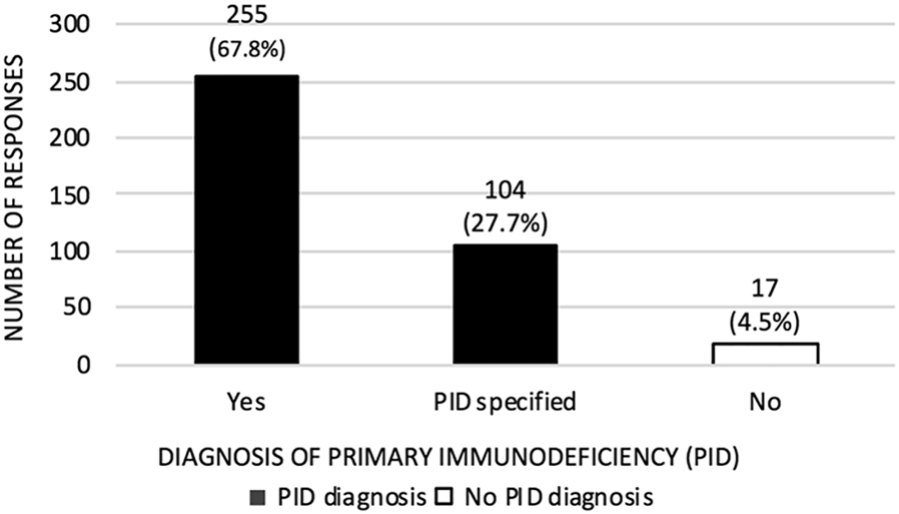
Respondents with diagnosis of primary immunodeficiency. Data from COVID-19 Vaccine Hesitancy Survey in Primary Immunodeficiency Patients, March 24 to April 7, 2021

A total of 449 individuals participated in the survey, with 448 respondents who answered and 1 who skipped the first question on the privacy and consent statement (Table 1). For all subsequent questions, at least 71 respondents skipped a question. There were eight questions targeted at all respondents—355–378 respondents answered each of these questions; the remaining four questions targeted subgroups of vaccinated, unvaccinated, and vaccine-hesitant respondents. Overall, there was a strong survey response rate of 39.7% (449 respondents among 1131 people targeted by email) relative to response rates for previous surveys by CIPO on a range of topics. Among 376 respondents, a large majority of respondents indicated they had a diagnosis of PID (67.8%) or specified the PID (27.7%); 4.5% indicated they had no PID diagnosis (Fig. 1).

**Table 1:** COVID-19 vaccine hesitancy questionnaire

Question	Answer choices	Responses	
		(%)	n =
I accept and have read the Privacy & Consent Statement	Agree	99.55%	446
	Disagree	0.45%	2
		Responses	448
		Skipped	1
		Total	449
Age	[Open]	Total	372
Date of birth	[Open]	Total	355
Gender	Female	75.4%	285
	Male	24.07%	91
	Non-Binary	0.53%	2
	Unknown/undecided	0%	0
	Other (please specify)	0%	0
		Total	378
Which of the following best represents your racial or ethnic heritage? (Choose all that apply)	Black, Afro-Caribbean, African-Canadian	0.84%	3
	Caucasian	92.42%	329
	East Asian or Asian	0.56%	2
	First Nations	0.28%	1
	French Canadian	2.81%	10
	Hispanic/Latino	0.84%	3
	Inuit	0%	0
	Metis	1.12%	4
	Middle Eastern or Arab Canadian	0.28%	1
	Non-Hispanic White or Euro-Canadian	6.46%	23
	Pacific Islander	0.28%	1
	South Asian	0.28%	1
		Total	356
Do you have a diagnosis of a primary immunodeficiency?	Yes	67.82%	255
	No	4.52%	17
	Please specify which one if known (ex. CVID, XLA etc.)	27.66%	104
		Total	376
Have you already received the COVID-19 vaccine?	Yes, 1st dose	19.68%	74
	Yes, 1st and 2nd dose	1.33%	5
	No, I have not had the COVID-19 vaccine	78.99%	297
		Total	376
If you answered *yes* to the previous question, which vaccine did you receive?	Moderna	7.63%	10
	Pfizer	38.17%	50
	Johnson and Johnson	0%	0
	AstraZeneca	8.4%	11
	I don’t know	6.11%	8
	N/A	39.69%	52
		Total	131
If you have not received the COVID-19 vaccine, do you plan on getting vaccinated when it is available to you?	Very likely	76.22%	282
	Somewhat likely	5.41%	20
	Somewhat unlikely	1.89%	7
	Very unlikely	2.43%	9
	Don’t know	10.00%	37
	No	4.05%	15
		Total	370
If you answered *unlikely, very unlikely, don’t know* or *no* to the previous question, please choose all that apply	I am concerned about long-term side-effects that are yet to be uncovered	50.41%	61
	I am not sure if there would be benefit as I might not have a response given my underlying immunodeficiency	56.20%	68
	I am not confident in the vaccine as it is too new and the process feels rushed	30.58%	37
	I have a history of allergies and am afraid of reacting to the vaccine	32.23%	39
	Even if the COVID-19 vaccine is available to me, I plan on waiting for more data being available on effectiveness and side-effects before deciding on whether I will get the vaccine	22.31%	27
	I am skeptical of the science behind the COVID-19 vaccine	16.53%	20
	I am skeptical of the medical system and the data regarding these vaccines	14.88%	18
	I do not believe in vaccination	1.65%	2
	I just do not want to get vaccinated	2.48%	3
	I already have or think I have contracted COVID-19	4.96%	6
	Other (please specify)	31.40%	38
		Total	121
Do you try to get the flu vaccine every year?	Yes	76.11%	274
	No	23.89%	86
		Total	360
If you answered *no* to the previous question, please choose all that apply	I am not aware I should get vaccinated yearly for influenza	7.27%	8
	My healthcare provider never discussed the importance of vaccination	8.18%	9
	I always get sick after the flu shot	17.27%	19
	I do not find the flu shot effective in preventing me from getting the flu	13.64%	15
	I just chose not to get vaccinated	16.36%	18
	Other (please specify)	46.36%	51
	N/A	15.45%	17
		Total	110
Did you receive the flu vaccine for 2020–2021	Yes	71.07%	258
	No	28.93%	105
		Total	363

Data from COVID-19 Vaccine Hesitancy Survey in Primary Immunodeficiency Patients, March 24 to April 7, 2021

*N/A* not applicable

Of 101 respondents who specified their PID, 71.3% reported having common variable immunodeficiency (CVID), which is often the most frequently diagnosed PID; 10.9% reported having hypogammaglobulinemia, and 6.9% had an immunoglobulin deficiency. A variety of other antibody disorders and immune disorders were reported in small numbers. Three non-specific responses were excluded from the estimations (Table 2).

**Table 2:** Immunodeficiencies among 101 COVID-19 survey respondents

Type of primary immunodeficiency disorder	Percentage (%)	Responses^*^
Common variable immunodeficiency (CVID)	72.3	73
Hypogammaglobulinemia	10.9	11
Immunoglobulin deficiency (IgA, IgA1, IgG, IgG2, IgG3, IgM)	6.9	7
STAT1 deficiency	2.0	2
B cell dysfunction	1.0	1
Chronic lymphocytic leukemia (CLL)	1.0	1
IPEX syndrome	1.0	1
Severe combined immunodeficiency disease (SCID) Omenn Syndrome *RAG1*	1.0	1
Secondary hypoglobulinemia	1.0	1
Secondary immunodeficiency	1.0	1
HLA-B27 autoimmune disease	1.0	1
Dysgammaglobulinemia	1.0	1

Data from COVID-19 Vaccine Hesitancy Survey in Primary Immunodeficiency Patients, March 24 to April 7, 2021

^*^Three non-specific responses were excluded from the estimations

*CLL* chronic lymphocytic leukemia, *CVID c*ommon variable immunodeficiency, *HLA-B27* human leukocyte antigen B27, *Ig* immunoglobulin, *IPEX* immunodysregulation polyendocrinopathy enteropathy X-linked, *RAG1* recombination activating 1 gene, *SCID* severe combined immunodeficiency disease, *STAT1* signal transducer and activator of transcription 1

**Fig. 2:**
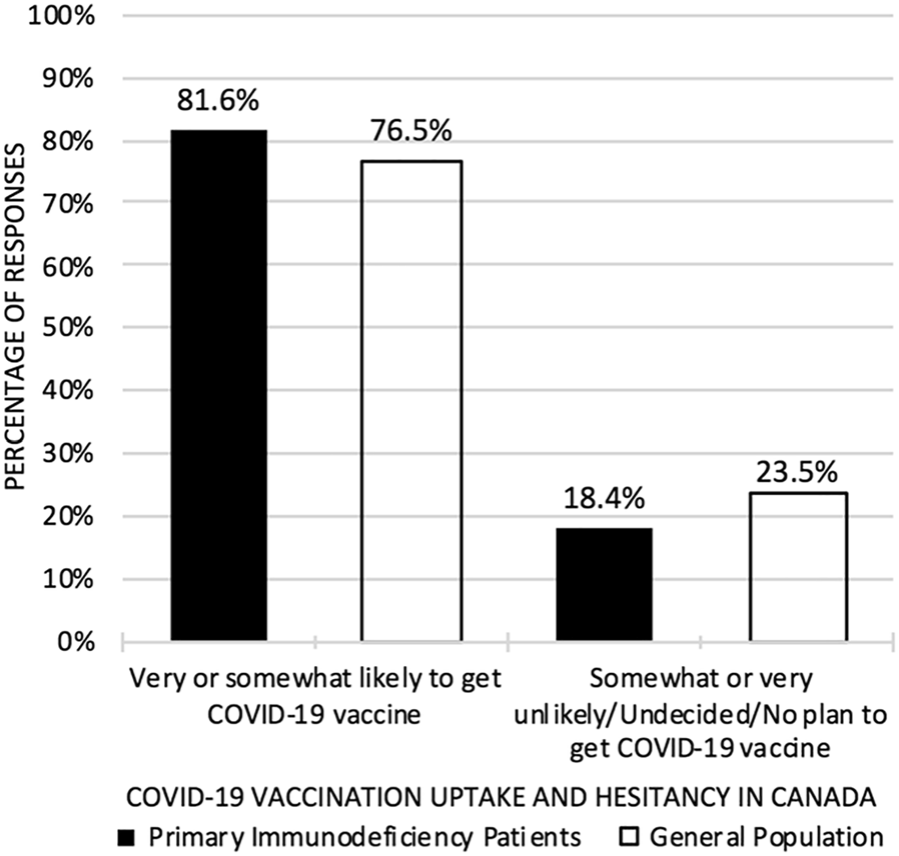
COVID-19 vaccine uptake and hesitancy among primary immunodeficiency patients compared to the general population. Data from COVID-19 Vaccine Hesitancy Survey in Primary Immunodeficiency Patients, March 24 to April 7, 2021. Data for general population extrapolated from Statistics Canada’s Canadians’ willingness to get a COVID-19 vaccine: Group differences and reasons for vaccine hesitancy [25]

At the time of survey, among 376 respondents, 21.0% of respondents had already received their first dose of the COVID-19 vaccine and 79.0% had not. Among 370 respondents who had not received the COVID vaccine, 302 respondents (81.6%) indicated they were very or somewhat likely to get vaccinated against COVID-19; and 68 respondents (18.4%) indicated they were undecided, somewhat or very unlikely, or not planning to get the vaccine (Fig. 2).

Vaccine hesitancy among 121 patients with PID was primarily due to uncertainty about benefit and immune response given an underlying immunodeficiency (56.2%), followed by concerns regarding the unknown long-term side effects of the COVID-19 vaccine (50.4%); pre-existing history of allergic reactions and fear of reaction to the vaccine (32.2%); newness of the COVID-19 vaccine and perception of rushed process (30.6%); as yet insufficient amount of data on effectiveness and side effects (22.3%); and, to a lesser degree, skepticism of the underlying science (16.5%) and/or of the medical system and available data on the COVID-19 vaccines (14.9%) (Table 3). Among 121 respondents who indicated their reasons for COVID-19 vaccine hesitancy, in addition to the survey’s answer choices, 38 respondents specified a range of concerns, including health-related concerns such as their immunodeficiency, autoimmune disorder, co-morbidities, immune response, apprehension or history of severe adverse events, and/or allergic reactions to vaccines. They also expressed safety and efficacy concerns, which included inadequate investigation of COVID-19 vaccine effects on those with medical comorbidities and risk of severe side effects such as VITT. Several respondents reiterated their uncertainty and skepticism. However, only 5 of the 38 respondents (13.2%) expressed having plans or intentions to consult with their healthcare provider about whether or not to get vaccinated.

**Table 3:** COVID-19 vaccine hesitancy among 121 primary immunodeficiency patients in Canada

Reason for COVID-19 vaccine hesitancy	Percentage (%)	Responses
Not sure of benefit and immune response given underlying immunodeficiency	56.2	68
Concerned about long-term side-effects that are yet to be uncovered	50.4	61
History of allergies and afraid of reacting to the vaccine	32.2	39
Not confident in the vaccine as it is too new and the process feels rushed	30.6	37
Plan to wait for more data being available on effectiveness and side-effects	22.3	27
Skeptical of the science behind the COVID-19 vaccine	16.5	20
Skeptical of the medical system and the data regarding these vaccines	14.9	18
Already have or think I have contracted COVID-19	5.0	6
Do not want to get vaccinated	2.5	3
Do not believe in vaccination	1.7	2
Other (please specify)^*^	31.4	38

Data from COVID-19 Vaccine Hesitancy Survey in Primary Immunodeficiency Patients, March 24 to April 7, 2021

^*^Other reasons specified were mainly related to past experiences of allergic or adverse reactions to other vaccines; underlying immune/antibody deficiency; and lack of data on vaccine effects in people with primary immunodeficiency disorders or other health conditions

**Fig. 3:**
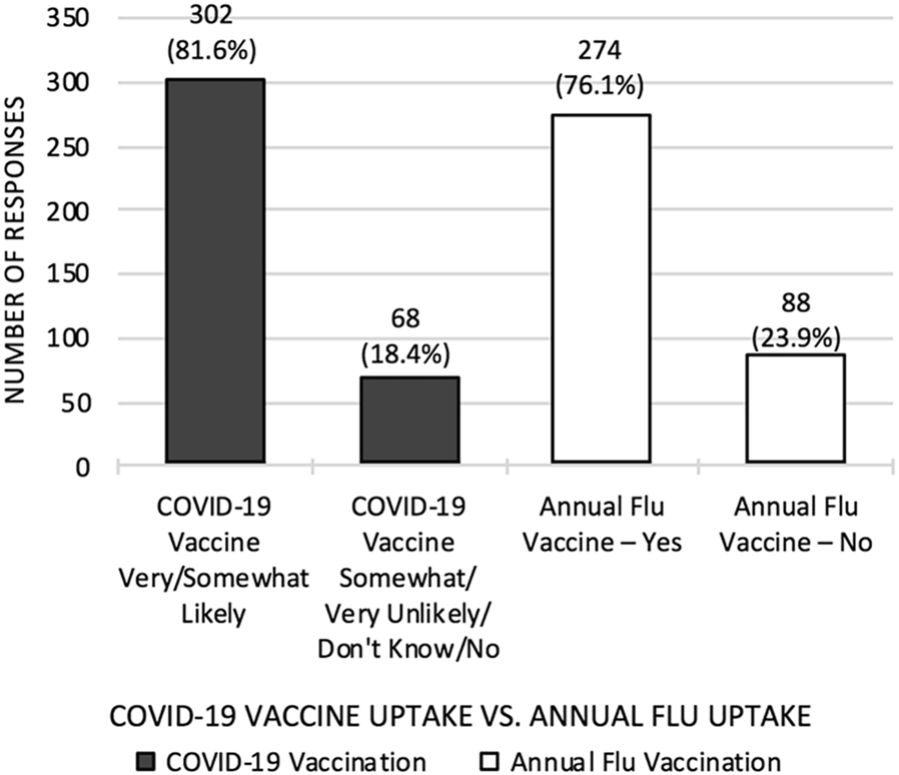
COVID-19 vaccine uptake vs. annual flu vaccine uptake. Data from COVID-19 Vaccine Hesitancy Survey in Primary Immunodeficiency Patients, March 24 to April 7, 2021

Regarding the influenza vaccine uptake, 76.1% of 360 respondents indicated that they try to get the vaccine every year; 71.1% of 363 respondents had received the vaccine for the winter of 2020–2021. Among 110 respondents who do not seek vaccination yearly, the more common reasons were sickness after the influenza vaccine (17.3%), perception that the influenza vaccine is not effective in prevention of influenza (13.6%), and personal choice (16.3%). Some reported being unaware that they should get vaccinated yearly for influenza (7.3%), and/or that their healthcare provider had never discussed the importance of vaccination (8.2%). In addition, 50 respondents specified a variety of other reasons including physician advice, previous allergic and/or adverse reactions to other vaccines or influenza vaccination, lack of immune response or perception thereof, reliance on herd immunity, unavailability of suitable vaccine, and/or uncertainty of the most suitable vaccine. Overall, the survey results indicated that respondents were slightly more inclined to get COVID-19 vaccination (81.6% of 370 respondents) compared to the influenza vaccination (76.1% of 362 respondents) (Fig. 3).

## Discussion

Our survey demonstrates that the majority of patients with PID were keen on getting vaccinated against COVID-19. These results from the PID community show a slightly higher inclination for COVID-19 vaccination and lower vaccine hesitancy than rates reported from a survey of Canada’s general population conducted by Statistics Canada in May and June 2020, prior to the availability of COVID-19 vaccines [14]. Among the small proportion of vaccine-hesitant respondents in our study, the most commonly cited reasons for being uncertain or not wanting to pursue vaccination included uncertainty regarding whether an immune response can be mounted due to their immunocompromised state and/or because the current use of immunosuppressant medications, lack of understanding of long-term side-effects of the vaccine, anxiety towards a possible reaction to the vaccine given a history of allergic reactions, the novelty and expediated process of the vaccine approval and the lack of long-term data availability. To a lesser extent, there were some reservations about the underlying science and trust of the medical system.

Due to immunocompromised state of the disorder, patients with PID are believed to endure a poor outcome with respect to morbidity and mortality should they contract COVID-19 [1, 3]. However, there conflicting evidence to suggest that this is limited to those with reduced IFN-1 signaling, a key cytokine for protective immunity against SARS-CoV-2, and those of younger age with PID [4–6]. In particular, younger males with PID are more likely to endure severe COVID-19 and require ICU admission [5]. Conversely, data emerging from Israel suggests that a small cohort of patients with PID who contracted COVID-19 had a mild disease course, with a third being asymptomatic. This has been attributed to a combination of patients following strict public health measures such as maintaining good hand hygiene, social distancing, mask wearing and severe isolation, as well as the notion that only specific immune pathways such as type I interferon signaling being the culprit for severe disease [15]. Regardless, vaccination against COVID-19 has been shown to decrease hospitalization and reduce mortality [16]. At this time, data regarding benefit of vaccination in patients with PID remain minimal but there is evidence to suggest that a partial, if not full, response can be obtained with the mRNA COVID-19 vaccines [7–9]. Canada’s NACI has recommended immunization against COVID-19 with any of the four approved COVID-19 vaccines, with a stronger predilection for an mRNA vaccine (Pfizer-BioNtech’s Comirnaty^®^, Moderna’s Spikevax^®^) due to their safety and the likelihood that the benefits would outweigh the risks of vaccination [10, 17]. Recently published cohort studies in Canada, the United States, and Israel have shown robust immunity against COVID-19 in some patients with antibody deficiency, and positive results with the use of COVID-19 vaccines to treat patients with PID [18, 19]. Observational studies in individuals with autoimmune conditions indicate that the frequency and severity of adverse events is comparable to that of those without autoimmune conditions. However, there is emerging evidence of a diminished or delayed immune response to COVID-19 vaccines in individuals with autoimmune conditions taking immunosuppressive therapies, with B cell depleting therapies and glucocorticoid therapy having the most profound impact [10, 20, 21].

Given the emergence of variants of concern, vaccine effectiveness may be decreased and subsequently additional vaccine doses may be necessary, particularly in the immunocompromised population where a diminished immune response to any of the authorized COVID-19 vaccines can occur [10]. Currently, the recommendation is a primary series of 3 doses with an mRNA COVID-19 vaccine should be preferentially offered to those that are immunocompromised. For these individuals who have previously received a 1- or 2-dose COVID-19 vaccine series (with a homologous or heterologous schedule using mRNA or viral vector vaccines), it is recommended that an additional dose of an mRNA COVID-19 vaccine should be offered [17, 22]. It remains yet to be determined whether vaccination against COVID-19 will become an annual requirement similar to the influenza vaccine.

Interestingly, our survey was consistent with the influenza vaccine, with the majority of patients with PID indicating that they had been vaccinated for the 2020–2021 season. Similar findings were found in a study on influenza vaccine hesitancy in the province of Quebec, in which the majority had received the influenza vaccine and only 32.2% of all respondents were identified as vaccine-hesitant (13.3% totally hesitant, 18.9% somewhat hesitant) [23].

**Fig. 4:**
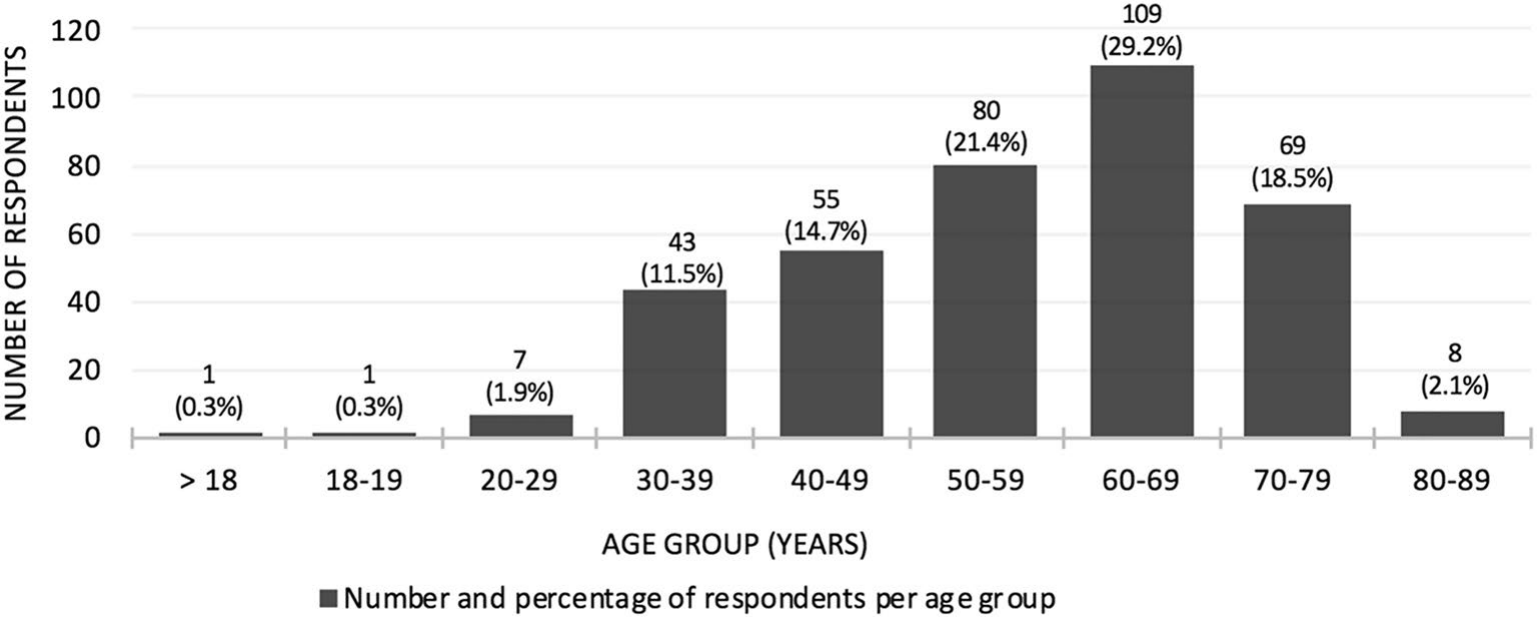
Age distribution of COVID-19 survey respondents. Data from COVID-19 Vaccine Hesitancy Survey in Primary Immunodeficiency Patients, March 24 to April 7, 2021 One respondent also provided their child’s age, therefore, while there were 372 respondents, age group calculations are based on 373 responses. One respondent gave a non-specific age (>18)

Our study is not without limitations as the data suggests possible under-representation of racial and ethnic populations. Among all respondents, the majority (92.4%) identified as being Caucasian with only a small percentage (13.8%) identified with at least 1 of 11 racial/ethnic groups (Table 4). This was an open-ended question that allowed respondents to select multiple options for racial/ethnic heritage with a very small number of respondents (6.1%) identifying with a least two different racial/ethnic groups. Canadian rapid review studies have identified some visible minority groups (including South Asian, Chinese, Black, Filipino, Latin American, Arab, Southeast Asian, West Asian, Korean, Japanese) to be at increased risk of exposure and increased risk of severe outcomes [10]. Outreach and strategies targeted at these populations would help ensure that they are informed about their COVID-19 risks and possibly encourage vaccine uptake. It is also worth noting that there was a lack of heterogeneity among PID type, as the majority of respondents had reported a diagnosis of CVID. In addition, the majority of patients with PID in our survey (71.3%) were 50 years of age and older. The lowest (2.2%) being those 18–29 years of age (Fig. 4). The low number of respondents in this age group may suggest a need for outreach targeted at this age group, particularly as a study from the United States has found up to a quarter of adults aged 18–25 were hesitant to accept a COVID-19 vaccine [24]. Furthermore, there was no data to assess for possible geographic, linguistic, or socioeconomic factors which may result in lack of access to a computer/digital device, the internet, or other technologies required to complete the survey. Incorporation of community kiosk or other modes of outreach for certain demographics could engage a larger number and greater diversity of people in the PID community. Finally, we were not able to capture data on where the vaccine-hesitant individuals reside, which could provide insight on geographic, socio-cultural, or other contexts that may be contributing to the hesitancy.

**Table 4:** Racial/ethnic background of COVID-19 survey respondents

Racial/ethnic heritage	Percentage	Responses
Caucasian	92.4	329
Non-Hispanic White or Euro-Canadian	6.5	23
French Canadian	2.8	10
Métis	1.1	4
Hispanic/Latino	0.8	3
First Nations	0.3	1
Middle Eastern or Arab Canadian	0.3	1
Pacific Islander	0.3	1
South Asian	0.3	1
Inuit	0.0	0

Data from COVID-19 Vaccine Hesitancy Survey in Primary Immunodeficiency Patients, March 24 to April 7, 2021

## Conclusion

In conclusion, this study illustrates the importance of ongoing patient outreach, education, and timely updates on the rapidly evolving scientific knowledge and evidence on COVID-19 relevant to the PID community in Canada, from clinical trials to real-world evidence and observational studies. Due to the heterogeneity of PIDs, COVID-19 vaccine guidance should include an advisory regarding health conditions and circumstances in which patients with PID should consult their primary care provider and/or a clinical immunologist and allergist for advice on COVID-19 vaccination. Informed consent should include discussion about the possibility that individuals who are immunocompromised may have a diminished immune response to any of the authorized COVID-19 vaccines. Active post-vaccination monitoring for efficacy in this population is particularly important. As the two main hesitancies among patients with PID getting vaccinated include concerns about the benefit and immune response to the COVID-19 vaccines as well as concerns regarding long-term vaccine effects, further research in these two areas is needed.

## Data Availability

The authors will consider making the relevant anonymized patient level data and material available on reasonable request.
